# From Policy to Action: Co‐Designing Research With Local Government to Advance Urban Greening

**DOI:** 10.1002/hpja.70164

**Published:** 2026-02-17

**Authors:** Carmel Williams, Yonatal Tefera, Veronica Soebarto, John Kandulu, Melanie Lowe, Jacinta Humphrey, Thami Croeser, Lara Daddow, Ben Willsmore

**Affiliations:** ^1^ Centre for Health in All Policies Research Translation South Australian Health and Medical Research Institute Adelaide South Australia Australia; ^2^ School of Public Health Adelaide University Adelaide South Australia Australia; ^3^ School of Architecture and Civil Engineering Adelaide University Adelaide South Australia Australia; ^4^ College of Business, Government and Law Flinders University Bedford Park South Australia Australia; ^5^ Centre for Urban Research, School of Global, Urban and Social Studies RMIT University Melbourne Victoria Australia; ^6^ ICON Science, Centre for Urban Research, School of Global, Urban and Social Studies RMIT University Melbourne Victoria Australia; ^7^ City of Salisbury Salisbury South Australia Australia; ^8^ City of Unley Unley South Australia Australia

## Abstract

**Issue Addressed:**

Urban greenspaces are widely recognised for their benefits to public health, the environment and climate resilience and are supported by strong policy commitments across federal, state and local governments. However, Australian cities continue to experience net greenspace loss, reflecting a gap between policy commitments and implementation. A key contributor to this gap is the systematic undervaluation of greenspace and urban trees within local government decision‐making, particularly where their health, environmental and social benefits are not adequately reflected in economic assessments. This study aimed to develop a locally relevant economic framework and tools to support local governments' efforts to operationalise their urban greening policy goals.

**Method:**

A co‐design methodology was applied over an 18‐month period with the City of Salisbury and the City of Unley local governments in Adelaide, Australia. The process included co‐design workshops and other stakeholder engagement activities, to co‐develop economic scenarios, iteratively refine an economic modelling framework and collaboratively develop a prototype decision‐support tool and associated communication resources.

**Results:**

The co‐design process enabled shared problem framing, led to the development of locally relevant economic scenarios, and refined model assumptions to reflect operational realities of the councils. Outputs included: (1) an economic modelling framework to quantify costs and benefits of urban trees; (2) a prototype Urban Greening Decision‐Making Tool and (3) tailored communication resources (including fact sheets, infographics and a policy brief).

**Conclusion:**

This case study demonstrates that bridging the policy–implementation gap requires more than producing evidence; it requires researchers to work in genuine, trust‐based partnership with practitioners. This involves approaching collaboration with humility and flexibility, listening to partners' priorities and co‐creating research and outputs.

## 1 Introduction

Urban greening is the conservation, restoration or creation of green infrastructure, in and around urban areas that benefit people, nature and the economy and the soils and water to support it. Greenspaces include parks, streetscapes, community gardens, bushland reserves and green corridors, as well as front and backyards on private land. A growing body of evidence shows that access to quality greenspace supports physical and mental health [1]. The pathways include strengthened social cohesion [2], mitigation of urban heat [3], enhanced biodiversity [4], removal of air pollutants [5] and improved climate resilience [6].

These health benefits can translate into measurable economic benefits. For example, reduced exposure to heat and air pollution is associated with lower healthcare costs and productivity losses [7], while improved mental and physical health is linked to reduced absenteeism and increased labour force participation. Urban cooling from tree canopy can lower building energy demand and household cooling costs [8], and improved urban greenspace is associated with higher property values and local economic activity [9]. Reflecting this evidence, governments have adopted urban greening policy targets, including tree canopy expansion goals [10, 11], climate adaptation plans [11] and health and wellbeing strategies that incorporate nature‐based solutions [12].

Despite ongoing government policy commitments and targets to increase urban greening, Australian cities continue to experience net greenspace loss. For example, Adelaide in South Australia is estimated to lose around 75 000 trees annually and has a tree canopy cover of approximately 17%, among the lowest for state capital cities in the nation [13]. This reflects a persistent gap between policy intent and implementation, particularly at the local government (hereinafter referred to as council) level.

This paper presents an Adelaide‐based case study of a co‐designed research project, titled ‘Economics of Trees’, which aimed to develop a locally relevant economic framework and tools to support councils' efforts to operationalise their urban tree planting policy goals. The economic framework focuses and was underpinned by the authors' previous comprehensive economic analyses for street trees [14].

Councils are key settings for preventive health action, with direct control over urban greenspace, one of the critical health‐promoting resources influencing population health, equity and climate resilience [15]. By demonstrating how economic frameworks can be co‐produced and mobilised to support urban greening, this study provides the health promotion community with a concrete example of how upstream, place‐based interventions can be operationalised in partnership with councils to achieve sustained public health impact.

## 2 Project Context

### 2.1 Urban Greening Policy and Governance in Australia

Urban greening is increasingly supported by policy commitments across all three levels of government in Australia. At the federal government level, national policy frameworks have begun to more explicitly recognise the role of nature‐based solutions in climate adaptation, urban resilience and public health. For example, the National Adaptation Plan [16] includes ‘investing in blue and green infrastructure and integrated catchment management to support urban cooling, climate resilience and flood mitigation in urban areas’. The National Urban Policy [17] also provides high‐level support for green infrastructure planning, biodiversity protection and community wellbeing outcomes linked to access to nature.

At the State government level, urban greening is embedded within land use planning legislation and climate‐related strategies. In South Australia, for instance, the Planning, Development and Infrastructure Act 2016 [18] establishes the framework for all spatial planning and development decisions and includes provisions for the establishment of an Urban Trees Fund to support the planting, maintenance and protection of trees. This is operationalised through the Planning and Design Code [19], which sets out specific planning policies aimed at increasing tree canopy cover, enhancing biodiversity and fostering more liveable neighbourhoods. These statutory tools are complemented by strategies and plans such as the Urban Greening Strategy, South Australia's first dedicated strategy to increase tree canopy [20], the South Australian Government's Climate Change Action Plan, which explicitly identifies urban greening as a priority action area [21] and the 30‐year Plan for Greater Adelaide, which aims to increase urban green cover by 20% by 2045 and achieve 30% tree canopy cover across metropolitan Adelaide by 2055 [22]. These policies, strategies and plans signal strong intent from the state government to embed urban greening as a core component of land use and infrastructure planning.

Councils carry the responsibility for managing community land and public assets, including the provision, management and maintenance of urban greenspaces on public land [23]. Many councils have developed their own Urban Forest Strategies, Open Space Plans and Tree Management Policies that set out specific greening targets and priorities. These documents often include commitments to increase canopy cover, improve equitable access to greenspace, and enhance urban forest diversity through local planning, development assessment and asset management processes.

At council level, these policy intentions are translated into practice through a combination of statutory planning instruments, strategic planning documents and ongoing asset management processes. Statutory controls, particularly development assessment undertaken under the Planning and Design Code, influence where and how trees and greenspaces are retained, removed, or required as part of new development and urban infill. These statutory mechanisms are complemented by council‐level strategic documents, such as Urban Forest Strategies and Open Space Plans, which articulate long‐term canopy targets, planting priorities and principles for equitable access to greenspace. Implementation is further shaped by asset management frameworks that govern the planting, maintenance, renewal and removal of trees and other green assets over their lifecycle, often within constrained operational and capital budgets. Together, these instruments define the practical decision‐making environment in which councils seek to deliver urban greening outcomes and highlight the need for locally relevant economic evidence to support prioritisation and investment decisions.

### 2.2 Project Background

The core idea of the ‘Economics of Trees’ project was conceived from the research team's prior collaborations with councils and state governments on urban greening initiatives, including the Healthy Parks Healthy People SA initiative [12] and the South Australian Green Infrastructure Action Plan [24]. These partnerships revealed firsthand the practical challenges governments face in implementing their greening policies and tree canopy targets. Initially, limited progress was attributed to a lack of evidence‐informed policy frameworks. However, as such frameworks were progressively developed and endorsed at both state and local government levels, attention shifted to understanding barriers to implementation.

A key contributor to this challenge is the systematic undervaluation of greenspace since most of the existing valuation frameworks and tools fail to comprehensively capture the full range of health, environmental and social co‐benefits [25]. Councils often rely on standard asset management and financial management processes that do not account for public benefit. This challenge is compounded by a structural imbalance in which the benefits of urban greenspace are largely public, while the costs of planting, maintaining and protecting trees are borne primarily by councils. These contribute to trees and greenspace being frequently framed as liabilities or maintenance costs rather than long‐term assets with co‐benefits across health, wellbeing, climate adaptation and biodiversity. Although previous initiatives have developed valuation resources and frameworks [26], there remains a lack of accessible, locally relevant tools that can help councils make a compelling business case for investing in new and existing greenspaces.

The ‘Economics of Trees’ pilot project was designed to address this challenge. The project was piloted in two local councils in the Greater Adelaide Metropolitan area in South Australia—the City of Salisbury and the City of Unley. The selection of these two councils was purposeful, as they have diverse communities, urban form and existing greenspace coverage. In 2021, the City of Salisbury had a population of 145 806 and ranked among the least advantaged 20% of local government areas in Australia (Index of Relative Socio‐Economic Disadvantage—IRSD score: 904). In contrast, the City of Unley, with a population of 38 641, ranked within the most advantaged 10% (IRSD score: 1067) [27]. While Unley has a much higher tree canopy cover (25%) than Salisbury (13%) [28], it faces a risk of losing it due to an increasing trend in urban infill development [29]. Both local areas are confronted by different challenges in retaining and expanding greenspaces, as well as preventing the loss of existing tree canopy and greenspace.

## 3 Methods

### 3.1 Theoretical Framework

The ‘Economics of Trees’ project employed a participatory co‐design approach [30], grounded in action research principles of knowledge co‐production and research–practice collaboration. Action research involves systematic enquiry aimed at improving the work practices of a certain discipline, usually with the involvement of practitioners from that discipline. In this study, we involved researchers and local councils and applied the Three Lenses Theory [31], bringing the scientific knowledge of the research team together with the political judgement and professional practices of the council partners to ensure that the project findings and outputs were fit for purpose, scientifically robust, reliable and acceptable to local governments, with the potential to be scaled across multiple Australian jurisdictions. The research team brought together expertise in economics, public health, urban planning, the built environment and public policy, supported by extensive experience in co‐design and research translation with government and policy actors.

### 3.2 Stakeholder Engagement and Co‐Design

The project was structured around multiple phases of engagement: (1) co‐framing of the research problem, (2) development and refinement of economic scenarios, (3) co‐design of a digital modelling tool and (4) co‐creation of communication outputs. The project was implemented over 18 months, between January 2024 and July 2025. The engagement process included planning meetings and a series of structured workshops, complemented by regular online meetings, collaborative document reviews and email exchanges.

A total of 11 engagement sessions were conducted, comprising eight face‐to‐face workshops and three online meetings. Participants included members of the research team, the project advisory committee, two council researchers and council staff from diverse departments such as planning, sustainability, city shaping, urban design, arboriculture, asset management, parks and open space management and community wellbeing and engagement. This iterative engagement process enabled continuous refinement of the research purpose, methodology and outputs in response to practitioner feedback, ensuring the project remained responsive to local contexts and fostered shared ownership.

### 3.3 Economic Modelling

The economic component of the project aimed to quantify the costs and benefits associated with different urban street tree planting and management scenarios. The modelling approach drew on principles from benefit–cost analyses (BCA), with outputs expressed in terms of net present value (NPV) and benefit–cost ratio (BCR). The BCA framework used in the project considered various benefits associated with urban street tree planting, including from flood mitigation, improved water and stormwater quality, air pollution removal, climate regulation, building energy use reduction, improved biodiversity, increased urban amenity and reduced heat‐related illnesses and mortality [14]. It compared outcomes under a ‘with project’ versus ‘without project’ scenario. The analysis used a 30‐year time frame, allowing for the greenspace to fully establish and deliver long‐term benefits.

### 3.4 Decision‐Support Tool and Other Resources

Drawing on outputs from economic modelling of urban street tree investments, the research team developed a prototype Urban Greening Futures Decision‐Support Tool. This tool is designed to address key operational and strategic needs identified during co‐design with council partners, specifically the City of Unley and City of Salisbury.

In addition to the decision‐support tool, the team co‐developed a suite of visual and written communication outputs in consultations with the councils, including a fact sheet, infographic, a final project report and a policy brief.

## 4 Results

### 4.1 Co‐Design and Engagement Process

Before the project commenced, the researchers had engaged with key stakeholders from both councils to clarify the issue and ensure the researchers understood the problem from the councils' perspectives. These initial discussions were critical and led to the project obtaining approval from both councils' chief executives. This high‐level mandate and endorsement played an important role, as it legitimised the investment of council staff time, the sharing of sensitive council information and helped to maintain engagement throughout the life of the project.

**FIGURE 1:**
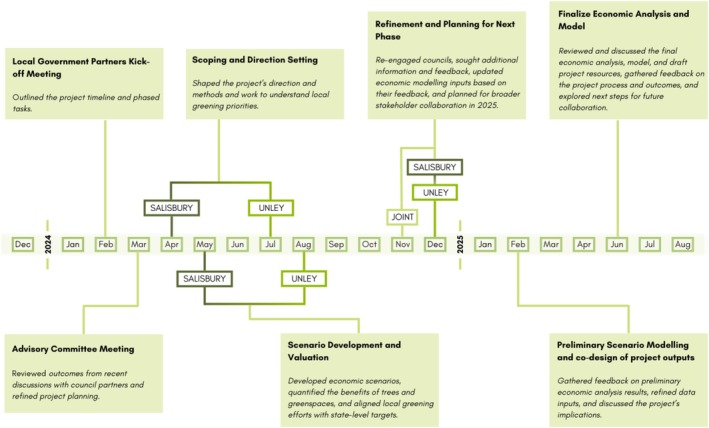
Overview of engagement phases and key project milestones.

Two key council staff joined the research team, which facilitated the wider engagement with other council staff. In addition, an advisory committee with representatives from industry partners, state government, local governments, NGOs and the research team was established to ensure the project could be expanded and upscaled in the future. Engagement and co‐design continued throughout the project, working through a series of online, face‐to‐face meetings and workshops, which led to the iterative development of the economic model and decision‐support tool. Figure 1 shows the critical timeframes of the overall engagement process. Details of these sessions, including their timing, participants, purposes and outcomes, are provided in Table S1.

#### 4.1.1 Co‐Framing the Research Problems

During the planning meetings, the research team worked with council partners to co‐frame the research direction. Drawing on experience from previous projects with local councils [12], the team recognised that council decision‐making is strongly shaped by financial viability assessments, within which urban greenspace and trees are often undervalued or deprioritised. The purpose of these planning meetings was therefore to understand why this undervaluation persists in practice and how it constrains the implementation of urban greening policies at the local level. This process ensured that the research focus remained grounded in the primary challenges councils were already grappling with, rather than abstract or purely academic objectives.

Across both councils, priority problems were consistently articulated in economic terms, including how to assess return on investment for trees, how to integrate urban forest strategies into internal decision‐making systems and how to justify greening investments to executive leaders and elected members. Councils emphasised the importance of developing robust but locally relevant economic scenarios and valuation methods that reflect not only environmental outcomes, but also social, health and amenity co‐benefits.

#### 4.1.2 Co‐Developing the Methods and Tools

As the project progressed into methodological development and tool design, the co‐design process deepened. An existing street‐tree economic valuation framework developed through the authors' previous research [14] was presented to the councils. This framework specified a set of benefit categories including climate regulation and urban cooling, air pollution reduction, stormwater management, heat‐related health outcomes, biodiversity and urban amenity. The co‐design process then focused on defining how these benefits could be modelled accurately and defensibly in the local context.

Council's feedback, communicated through both workshops and email exchanges, was critical in shaping the core assumptions of the BCA model. The consultation process was structured around ensuring the model's parameters and scenarios were grounded in local operational realities.

Table 1 describes the key model parameters and the feedback provided by council participants during consultation. In summary, council staff members challenged simplistic and uniformly applied urban greening targets. Their input prompted a shift in focus toward grounding the ‘Realistic’ and ‘Optimistic’ scenarios in practical local constraints and implementation realities. For example, feedback highlighted that while an ‘Optimistic’ 20% tree increase for Salisbury sounded positive, it was important to consider budget and planting capacity. Conversely, for Unley, given its high rates of urban infill and existing 25% canopy, stakeholders questioned if a ‘Realistic’ scenario might involve a net loss, even with proactive planting. This feedback ensured the final scenarios reflected the distinct institutional and policy dynamics of each municipality. Similarly, council staff identified that a uniform ‘cost‐per‐tree’ was inaccurate and pushed for a more granular, lifecycle‐based cost structure. Discussions focused on specifics, such as differentiating the high‐intensity formative care in years 1–5 from later routine inspections and factoring in end‐of‐life removal costs or the probability of tree failure. This feedback led the research team to source detailed, context‐specific cost data directly from each council for tree establishment, maintenance and removal.

**TABLE 1:** Key model parameters, definitions, consultation issues and final modelling assumptions for the context‐sensitive benefit–cost analysis of street trees in Salisbury and Unley.

Model parameters	Definition/assumption used in the model	Issues raised during consultation	Final decision/how handled in the model
Cost per tree	Total present value cost (TPVC) per tree includes establishment (purchase, planting, cage), maintenance (formative pruning, irrigation, inspection, routine maintenance), surgery/infrastructure damage mitigation, removal and replacement costs over 30 years.	Councils highlighted high uncertainty and variability in field service costs, frequency of visits, powerline pruning and infrastructure damage; noted that generic cost figures may not reflect local asset management practices.	Used council‐supplied ranges for each cost item with beta distributions; incorporated parameter uncertainty via Monte Carlo simulation; treated TPVC as a probabilistic distribution and reported means and percentiles; updated unit costs in consultation with Salisbury and Unley to better reflect local conditions.
Tree species selection	Representative palette of common street tree species for each council (Salisbury: Callery pear, Jacaranda, SA Blue gum, Queensland box, Bottlebrush, Claret ash, Coral gum; Unley: Snakebark maple, Watergum, Tuckeroo, Bottlebrush, Claret ash, Coral gum, Ginkgo). Tree size and structural attributes (height, canopy area, trunk girth, time to maturity) used to derive Tree Premium Multipliers (TPM) for amenity, carbon and stormwater.	Need to reflect ‘right tree, right place’ and differences in performance by species (e.g., cooling, roots, maintenance); concern about over‐generalising benefits across species and councils.	Modelled species explicitly and used species‐specific TPMs to scale benefits; scenario results aggregated over a representative species mix for each council; highlighted species‐level BCRs (figures on BCR by species) to inform species selection rather than assuming a uniform tree type.
Tree lifespan	Economic analysis period set to 30 years, allowing trees to establish and deliver key benefits; mortality and removal modelled via probability of tree removal (0.01–0.05 per year) and replacement only after year 5; end‐of‐life removal costs included in TPVC with removal assumed between years 20–30 for damaged/failed trees.	Questions about whether 30 years adequately captures full biological lifespan and long‐lived species; concern that late‐life benefits beyond 30 years are omitted, making analysis conservative.	Adopted 30‐year ‘effective economic lifespan’ consistent with infrastructure evaluation guidelines; treated mortality and replacement probabilistically; acknowledged that this likely underestimates long‐lived tree benefits and flagged results as conservative.
Canopy cover (%)	Baseline canopy: Unley ≈30% and Salisbury ≈19% at LGA scale; scenarios model linear changes in street tree population (and implied canopy) relative to 2025 baseline: Salisbury BAU −10%, Pessimistic −25%, Realistic +10%, Optimistic +20%; Unley BAU/Pessimistic −19%, Realistic −9%, Optimistic +1% by 2050.	Councils emphasised that canopy cover is constrained by limited verge/road space and infill, particularly in Unley; concern that city‐wide canopy targets do not reflect local planting opportunities.	Modelled changes in street tree numbers (proxy for canopy) linearly over time for each scenario; explicitly acknowledged Unley's limited scope for further canopy expansion; used per‐tree NPVs scaled by net incremental trees rather than prescribing a fixed canopy percentage target.
Planting density	Model implicitly assumes one tree per street planting position; changes in tree population (per scenario) represent net additions/removals, not explicit spacing; planting density therefore embedded in council street design and species selection rather than modelled as a separate variable.	No explicit planting density parameter was raised; focus was on total tree numbers, available sites and conflicts with infrastructure/parking.	Treated planting density as embedded in council street design standards; modelled scenarios in terms of net tree counts rather than trees per hectare; planting density not parameterised separately and is effectively ‘Not specified’ as an explicit model input.
Maintenance costs	Includes formative maintenance (first years), irrigation, inspection (field service) and inspection frequency, routine annual maintenance, plus surgery/infrastructure‐related costs where relevant; each cost parameter specified as a range with beta distribution (e.g., formative 27–40 AUD/tree, irrigation 79–119 AUD/tree, routine maintenance 6.56–8.20 AUD/tree/year).	Councils noted large variability in actual maintenance due to species, site, powerlines and extreme weather; concern that underestimating maintenance would weaken credibility, but overestimating could bias against trees.	Used council‐supplied ranges and beta distributions to represent uncertainty; included sensitivity analysis (tornado diagram) to show influence of maintenance parameters on TPVC; adopted conservative (higher) maintenance ranges where in doubt to maintain credibility.
Time horizon	30‐year analysis period (2025–2055) for costs and benefits, intended to allow for establishment and delivery of medium‐ to long‐term ecosystem services; some benefit modules (e.g., carbon and energy) use internal sub‐structures (e.g., S‐curve growth, maturity from year 10) within this 30‐year window.	Stakeholders queried whether 30 years is sufficient for large trees, and noted that many benefits continue accumulating well beyond this horizon.	Adopted 30 years to align with government infrastructure guidance and to keep analysis tractable; noted explicitly that this underestimates very long‐term benefits and framed reported NPVs/BCRs as conservative lower bounds.
Discount rate	Central discount rate of 7% per annum, with uncertainty represented by a beta distribution over 6%–8%; applied consistently across all cost and benefit streams to convert future annual values into present‐day equivalents.	Concern that standard public‐sector discounting heavily down‐weights long‐term tree benefits (maturity 15–20+ years), biasing comparisons in favour of short‐lived or immediate‐return infrastructure.	Used 7% as base case to align with government practice and maintain policy relevance; represented discount rate as a distribution in Monte Carlo analysis; recommended exploring lower or alternative discount rates in future work and flagged current results as conservative with respect to long‐term greenspace benefits.

*Note:* Data inputs for all model parameters were compiled from a combination of: (i) detailed establishment, maintenance, removal and replacement cost schedules provided by asset and arboriculture staff at the Cities of Salisbury and Unley; (ii) published peer‐reviewed studies and government guidelines for discounting and valuation of ecosystem services and (iii) street tree population trajectories (and associated implied canopy cover and planting intensity) co‐developed through iterative workshops with council representatives to reflect locally realistic Business‐as‐Usual, Pessimistic, Realistic and Optimistic scenarios.

The co‐design process also refined the model's inputs. For example, the team's initial species lists were revised to match the councils' approved planting palettes, which are based on local suitability. Feedback, for instance, questioned how the model would account for the different benefits of a Jacaranda versus a Callery Pear, given that council selection is driven by climate resilience, infrastructure constraints and biodiversity goals. This feedback was instrumental in the development of the ‘Tree Premium Multiplier’ (TPM) framework to assess species‐specific performance. Finally, stakeholders challenged key model assumptions, particularly the timing of benefit accrual. They correctly pointed out that a newly planted sapling does not provide the same benefits as a mature tree, questioning how the 30‐year benefit timeframe would be staged to reflect maturity. This feedback directly informed the use of S‐curve growth models and the assumption that many benefits begin accruing only after an establishment period.

Based on this iterative feedback, the research team refined the model parameters. This included tailoring the species mixes, incorporating detailed, non‐uniform cost data and revising outputs to reflect plateauing tree populations over the 30‐year horizon, particularly in space‐constrained areas like Unley. The scenarios were thus co‐developed to align with each council's unique urban form, planning constraints, budget, community profile and political priorities.

### 4.2 Outputs of the Co‐Designed Project

Key outputs of the project included a prototype decision‐support tool, fact sheets, infographics and technical reports. Here, we describe these key outputs and strategies used to disseminate these resources and share the project's findings with key stakeholders.

#### 4.2.1 Building a Fit‐for‐Purpose Decision‐Support Tool

The economic analysis by Kandulu and Soebarto [14] was translated into a prototype ‘council‐friendly’ Urban Greening Futures Decision‐Making Tool, which models the costs and benefits of various urban layouts and tree planting scenarios. The tool provides councils with an intuitive digital interface, purpose‐built for accessibility and ease of use in real‐world planning workflows. Users can:

- Input location‐specific data relevant to their municipality, ensuring analyses are contextually grounded.
- Select from co‐designed greening scenarios, ranging from baseline/pessimistic projections (such as projected declines in tree population with no new investment) to realistic and optimistic scenarios representing various levels of strategic greening intervention.
- Obtain quantitative estimates of costs, benefits, NPV and BCR with dynamic calculations powered by backend modelling frameworks.
- View disaggregated benefit categories, including health‐related benefits, carbon sequestration and storage, stormwater management (flood mitigation), air pollution reduction, energy and emissions savings, and avoided heat‐related morbidity. These outputs allow councils to identify, justify and communicate the multi‐dimensional value of urban greening.
- Support internal budget justification and strategic planning, equipping staff and decision‐makers with evidence‐backed analyses to inform council submissions, management discussions and cross‐departmental engagement.

While initially tailored for the operational context and data structures of the City of Unley and Salisbury, the system framework and backend modelling were designed for future scalability. This ensures adaptability as the tool is extended to support a broader range of Australian councils, with potential for customisation to meet diverse regional needs and evolving greening policy priorities.

#### 4.2.2 Other Resources and Outputs

**FIGURE 2:**
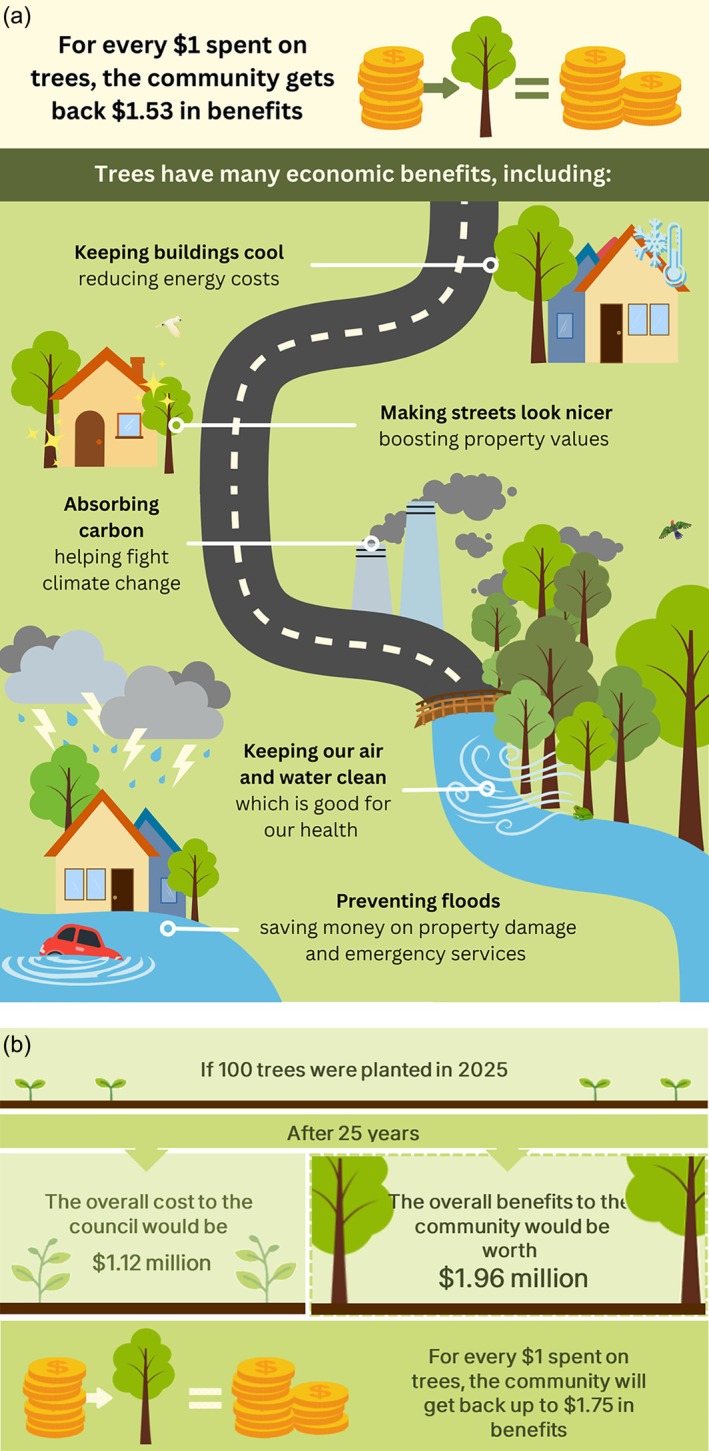
(a) Excerpt from the project fact sheet, and (b) accompanying infographic.

In addition to the decision‐support tool, the project produced a suite of co‐developed resources to support policy translation, stakeholder engagement and broader dissemination. These include a combined fact sheet and infographic (see Figure 2) that distil the project's key processes and findings into a clear, accessible format designed for use across government, academia, research, industry and community organisations [32].

A comprehensive final project report [33] was also developed to synthesise the findings, serving both to inform broader policy and research agendas and to demonstrate accountability to project funders through transparent documentation of outcomes and resource use. A policy brief tailored to senior management and elected members was also prepared [34]. Together, these digital and visual resources strengthen communication, advocacy, and knowledge exchange among policymakers, council officers, urban planners and community stakeholders. They serve as practical tools to inform and enhance current and future urban greening strategies.

### 4.3 Dissemination of Project Findings and Outputs

A key component of the project was the strategic and practice‐oriented dissemination of both findings and co‐developed outputs, designed to ensure broad accessibility, relevance and impact across multiple stakeholder groups. Importantly, the project went beyond traditional academic dissemination by actively engaging with local government processes and tailoring outputs to support real‐world decision‐making.

A range of dissemination strategies were employed to reach and resonate with diverse audiences, including council staff, elected members, community stakeholders and senior executive teams.

The research team worked closely with council partners to co‐identify key dissemination opportunities that aligned with internal planning cycles, policy milestones and advocacy needs. This included direct presentations and discussions with decision‐makers, such as the targeted briefing provided to the City of Salisbury's senior management team, where findings were discussed in the context of upcoming infrastructure and greening priorities.

## 5 Discussion: Implications for Practice and Policy

This project offers a grounded example of what it takes to embed a co‐design approach in research with local government and why this approach matters, particularly in tackling problems that involve a complex network of stakeholders, regulations and competing agendas. Our experience shows that genuine co‐design is not an add‐on; it is a foundational process that must begin before research purpose was finalised and continue through to implementation and dissemination.

For health promotion researchers working in policy‐relevant and practice‐facing fields, co‐design requires a fundamental shift in how research is conceived and conducted. It demands that researchers relinquish some control, remain open to iterative changes and balance academic rigour with the pragmatic realities and constraints of local government systems. This means engaging early, listening deeply and being prepared to adapt established methodologies to align with council workflows, systems, political priorities and timeframes.

At the heart of the project was the focus on engagement and shared decision making with the council partners. As Oliver and Cairney argue [35], influencing policy is not just about delivering robust evidence, but about understanding policy processes, building trusting relationships, demonstrating political judgement, and adapting to decision‐making environments. Our approach mirrored these principles: rather than developing a research agenda independently and seeking partner endorsement later the project was shaped through continuous dialogue and co‐framing from the outset.

For example, the co‐design process began before the project formally commenced. The research team were committed to delivering high quality research but equally committed to ensuring the project would be useful for partners. Prior to the research commencing, members of the research team met with local government partners and tested the research purpose and protocol with partner and their executives of both councils. This saw the research purpose modified to better meet the interest of local government. This early engagement also laid the foundation for trust, open dialogue and joint problem‐solving when challenges inevitably emerged.

Open dialogue and regular communication enabled the research team to gain a detailed understanding of the context in which the two local government partners were operating. Each partner brought different priorities, constraints and institutional logics, reinforcing the importance of Head's three lenses of evidence‐informed decision‐making [31]. Navigating these lenses required the research team to move beyond rigid methodologies and adopt a flexible, relational and pragmatic approach; this has been noted as a key to success in European greening innovation ‘living labs’, where rigid timeframes, budgets and project outputs tended to limit the scope for effective, evolving collaborations between practitioners and research teams [36].

For example, as local government partners became more engaged, they provided increasingly detailed and context‐specific information on tree planting, maintenance costs and canopy projections. These data inputs, though often iterative, allowed the economic analysis to reflect real‐world cost structures and implementation pathways. Incorporating these updates required the research team to continuously adjust scenarios and assumptions, resulting in additional workload and timeline extensions. However, this flexibility increased the credibility, relevance and legitimacy of the final modelling outputs, core attributes of impactful research in policy settings. Such sustained engagement exemplifies the practical guidance of Oliver and Cairney, to engage routinely, flexibly and humbly [35].

Importantly, the co‐design extended beyond research purpose and method development to include product development and dissemination strategies. For instance, a greater emphasis was placed on tailoring outputs for elected members and senior management teams, who play key roles in internal advocacy and budget decisions. This co‐ownership of outputs increased the likelihood that the tools would be used, not just admired.

Overall, the approach adopted throughout this project exemplifies key principles highlighted in the research translation literature [31, 35]. It reflects the ‘professional practice’ and ‘political judgement’ lenses described by Head [31], and responds directly to Oliver and Cairney's recommendations [35], notably the importance of making research relevant and readable and the need for researchers to reflect continuously on whether, how and why they engage.

## 6 Conclusion

This study demonstrated that translating evidence into impact, particularly within local government settings, requires more than technical expertise; it demands a fundamental mindset shift. Researchers must approach collaboration with humility, actively listening to the concerns, priorities and constraints of their practice partners. Rather than imposing pre‐defined agendas, the research process must be co‐created and co‐delivered, shaped by a shared commitment to producing outcomes that are both academically robust and practically useful. Flexibility, responsiveness and mutual respect are essential to building the trust‐based relationships that underpin meaningful collaboration, and ultimately, to achieving positive and lasting impact for partners and the communities they serve.

## Funding

This work was supported by Adelaide University; National Health and Medical Research Council (NHMRC), 2008937 and AXA Research Fund.

## Ethics Statement

Research Ethics approval was obtained from the Human Research Ethics Committee at Adelaide University, HREC number H‐2024‐063.

## Conflicts of Interest

The authors declare no conflicts of interest.

## Supporting information


**Table S1:** An overview of the engagement process.

## Data Availability

The data that support the findings of this study are available on request from the corresponding author. The data are not publicly available due to privacy or ethical restrictions.
