# Supplementary material for: From Policy to Action: Co‐Designing Research With Local Government to Advance Urban Greening
Source: Health Promot J Austr. 2026 Feb 17;37(2):e70164. doi: 10.1002/hpja.70164 (PMC12910417; doi:10.1002/hpja.70164)
Supplement: Supplementary file 1 — Table S1: An overview of the engagement process. [file HPJA-37-0-s001.docx]

Table S1: An overview of the engagement process.

| Description | Dates | Participants | Purpose | Key Outcomes |
| --- | --- | --- | --- | --- |
| Local Government Partners Kick off Meeting | February 2024 | Research team and representatives from participating councils | To outline the project timeline and phased tasks, and to plan the introduction of scenario-based workshops with each participating council. | Confirmed the overall project timeline and preliminary dates for scenario development workshops.  Agreed on the role and membership of the project advisory committee.  Confirmed the role of council partners in selecting and inviting additional council representatives to participate in the project. |
| Advisory Committee meeting | March 2024 | Research team, representatives from industry partners, local and state government, and NGOs | To review outcomes from recent discussions with council partners, refine project planning. | Project planning refinement. |
| Scoping & Direction Setting | Salisbury: April 2024  Unley: July 2024 | Council staff representing a broad range of departments including planning, sustainability, city shaping, urban design, arboriculture, asset management, parks and open space management, and community wellbeing and engagement | To shape the project’s direction and methods by identifying key planning and valuation challenges, and to understand local greening priorities within each participating council’s context. | Identified example scenarios to be used as case studies for the economic analysis, tailored to each council’s local context and community needs.  Discussed key priorities, opportunities, and challenges related to urban greening within each council.  City of Salisbury emphasised feasibility, community behavior, and the challenges of demographic shifts.  The City of Unley emphasised economic modelling, policy gaps, and the need to shift public perception of the economic value of trees. |
| Scenario Development & Valuation | Salisbury: May 2024  Unley: August 2024 | Council staff representing a broad range of departments including planning, sustainability, city shaping, urban design, arboriculture, asset management, parks and open space management, and community wellbeing and engagement | To develop economic scenarios (BAU, Pessimistic, Realistic, Optimistic), quantify the benefits of trees and greenspaces, and align local greening efforts with state-level targets. | Developed economic scenarios by selecting case studies for street trees, private land, and public spaces to inform the analysis.  Compiled a “data shopping list” identifying key modelling inputs required from each council. |
| Refinement & Planning for Next Phase**^^[[1]](#footnote-1)^^** | Joint Planning: Nov 2024  Salisbury: Dec 2024  Unley: Dec 2024 | Council staff representing a broad range of departments including planning, sustainability, city shaping, urban design, arboriculture, asset management, parks and open space management, and community wellbeing and engagement | To re-engage councils, seek additional information and feedback on proposed scenarios, update economic modelling inputs based on their feedback, and plan for broader stakeholder collaboration (e.g. SA Planning Commission) in 2025. | Refined economic scenarios using updated figures provided by councils.  Ensured the model’s accuracy and relevance to local contexts and conditions. |
| Preliminary Scenario Modelling and co-design of project outputs | February 2025 | Joint workshop between researchers and  representatives from both councils | To gather feedback on preliminary economic analysis results, refine data inputs, and discuss the project’s implications and the types of resources needed to support councils in applying the model. | Encouraged councils to share additional data to enhance the model’s local relevance.  Co-identified a suite of project outputs, resources, and tools to support councils in applying the model.  Refined the penultimate version of the economic model based on council feedback and updated inputs. |
| Finalise economic analysis and model | June 2025 | Joint workshop between researchers and  representatives from both Salisbury and Unley council | To review and discuss the final economic analysis, model, and draft project resources, gather feedback on the project process and outcomes, and explore next steps for future collaboration. | Final economic analysis and model endorsed by participating councils.  Agreed on the purpose and target audiences for draft project resources.  Outlined plans for developing a locally relevant decision-support tool.  Secured ongoing commitment from councils to scale up the project in the future. |

1. Online meetings [↑](#footnote-ref-1)
